# The Treatment of Syncope

**Published:** 1890-03

**Authors:** 


The Treatment of Syncope.—In the treatment of syncope, the first step is to place the patient in a recumbent position flat on the back, with the head low. The clothing should be loosened around the neck and body, the access of fresh air should be permitted, and to this end persons should be kept at a distance. Diffusible stimulants, as aromatic spirits of ammonia, and brandy or whisky, should be administered; or strong ammonia may be inhaled. Cold water may be dashed in the face, the respiration being thus excited, and in turn the heart caused to beat. If recovery ensues, the heart's beat becomes more distinct, the pulse reappears at the wrist, and consciousness slowly returns. It is only in cases where the heart is too badly damaged, as where there is fatty metamorphosis of its muscular fasciculi, or its valves are badly diseased, or where too much blood is drawn off, that resuscitation fails to take place.—Dr. Tyson, in the *University Medical Magazine.*

